# Antibiotic misuse among university students in developed and less developed regions of China: a cross-sectional survey

**DOI:** 10.1080/16549716.2018.1496973

**Published:** 2018-08-22

**Authors:** Dandan Peng, Xiaomin Wang, Yannan Xu, Chenhui Sun, Xudong Zhou

**Affiliations:** School of Public Health, Zhejiang University, Hangzhou, PR China

**Keywords:** University students, antibiotic use, regional disparity

## Abstract

**Background**: Antimicrobial resistance (AMR) is a great threat to public health. The primary cause of AMR is human antibiotic misuse. Little is known about regional differences of antibiotic misuse behaviours in China.

**Objectives**: To explore the antibiotic misuse behaviours among university students in western and eastern China and find out the regional differences.

**Methods**: Participants were recruited from universities in less developed Guizhou Province and developed Zhejiang Province using a cluster random sampling method. A self-administered questionnaire was designed to collect data, and the χ^2^ test and logistic regression were adopted to assess the associations between region and antibiotic misuse behaviours.

**Results**: A total of 2073 university students from Guizhou and 1922 from Zhejiang completed questionnaires. Students in Guizhou had lower household income, parents’ education, and urban residence proportion than those in Zhejiang. Compared with those in Zhejiang, students in Guizhou had higher antibiotic use prescribed by doctors (79.8% vs 56.2%) and self-medication with antibiotics (33.0% vs 16.1%). Students in Guizhou were more likely to buy over-the-counter antibiotics without prescriptions (73.9% vs 63.4%), ask for antibiotics from doctors (21.4% vs 15.6%), and use antibiotics prophylactically (29.9% vs 15.7%). Adjusted models showed that the less developed region was significantly associated with higher antibiotic misuse behaviours.

**Conclusions**: Misuse of antibiotics by well-educated young adults was very high in two regions but most serious in the less developed one. Campaigns are urgently needed to promote appropriate antibiotic use especially in less developed regions.

## Background

The advent of antibiotics is considered one of the most critical advents in medical science, and has saved millions of lives over the past 100 years [1]. However, the effectiveness of antibiotics against infectious diseases has been dramatically undermined as a result of emerging antimicrobial-resistant bacteria. Antimicrobial resistance (AMR) is among the greatest threats to global health, and represents a major contributor to rising healthcare costs worldwide [2–4]. The review on antimicrobial resistance in 2014 estimated that current annual mortality attributable to AMR is 700,000, and may rise to 10 million by 2050 if action is not taken to reduce inappropriate use of antibiotics [2]. The routine and inappropriate use of antibiotics for self-limiting illnesses, such as flu, cold, sore throat, and diarrhoea, is recognized as the most important factor responsible for increasing antimicrobial resistance [2,4,5].

In China, antibiotics misuse is pervasive [6,7], leading to very high and growing rates of AMR in both hospital and community-acquired infections [8–10]. A review of antibiotic use in China demonstrated that 80% of inpatients and 50.3% of outpatients were prescribed with antibiotics, and as many as 75% of patients with seasonal influenza were treated with antibiotics [11]. With one-fifth of the world’s population living in China, this is a serious global concern. The spread of AMR in China is also facilitated by high population mobility, massive rural–urban migration, and increasing foreign travel in recent years [3].

The massive levels of antibiotic misuse for self-limiting diseases can be attributed to factors on both the supply and demand sides in the Chinese health system. On the supply side, health providers relied heavily on drug sales to make profits, which inevitably resulted in overprescribing of antibiotics by hospitals and community health facilities [3]. Loose regulations on the sale of over-the-counter antibiotics have also led to easy access to antibiotics in pharmacies [12]. On the demand side, a lack of knowledge on approriate antibiotic use in the general public has resulted in antibiotic misuse behaviours, such as self-medication with antibiotics and keeping leftover antibiotics at home [13]. The impact of the factors listed above on antibiotic misuse behaviours varies between the more and less developed regions of China, due to the differing levels of social and economic development and governance capabilities. To date, however, only a small number of studies have examined the disparities in antibiotic misuse behaviours between rural and urban settings, rather than between more and less developed settings.

In order to address this gap, the current study aims to compare antibiotic misuse behaviours among university students in a less developed western province and a more developed eastern province in China and to explore the association between socio-economic factors and antibiotic misuse behaviours.

## Methods

### Study settings and participants

This study is a part of a large national cross-sectional survey regarding antibiotic misuse behaviours among university students in China. Among the six sampled provinces, we selected the western province, Guizhou, and the eastern coastal province, Zhejiang, with greatly differing levels of social and economic development. The GDP per capital of Guizhou was 29,847 RMB (4,592USD) and Zhejiang 77,644 RMB (11,945USD), ranked 29th and 5th, respectively, among the 31 provinces of China in 2015 [14]. In each of the two provinces, a multi-disciplinary university was selected: Guizhou University and Zhejiang University.

### Instrument

The questionnaire was comprised of two sections: socio-demographic characteristics of participants and their healthcare-seeking behaviours related to antibiotic use. Questions regarding socio-demographics collected information including gender, age, education level, major, household income per month, and parents’ medical background and their education level. The behaviours included *self-treatment with antibiotics*, *antibiotics use prescribed by doctors*, *asking for antibiotics from doctors in the past month*, *buying antibiotics without prescriptions*, *keeping antibiotics in dormitory or home*, and *taking antibiotics prophylactically in the past year*. The questions on antibiotic use behaviours were adapted for the Chinese setting from previous studies [15,16]. Students were also asked to state the chemical or brand names of antibiotics they had used.

### Data collection

The data for this study, collected from September to November 2015, included 3995 university students drawn from the two participating universities. The electronic questionnaire tool Wen Juan Xing (Chinese Survey Monkey: https://www.sojump.com) was used to conduct the survey. A cluster random sampling method was adopted. Permission was initially obtained from authorities at each university to conduct the survey. The aim was to achieve a sample size of roughly 1800 students across a range of disciplines, including science, social science/the humanities, and medicine, at each university [17]. The class timetable on the main campus was obtained at each university the day before the survey was administered. The classes (25 to 35 classes in each university) were randomly selected and all students attending these classes were included. At each university, three investigators approached teachers, explained the aim of the survey, and asked for permission to speak to students before the class began. No teacher refused. The investigator then explained the aim of the survey to the students, disseminated the printed QR code (Quick Response Code) of the electronic questionnaires, and explained to students how to complete the electronic questionnaire. Over 95% of the students in the selected classes completed the questionnaire. A gratuity of 3RMB (0.5USD) was paid via smartphone to all the students who completed the questionnaire.

### Data management and analysis

A chi-square test was conducted to compare the socio-demographic characteristics and antibiotic misuse behaviours between students in Guizhou and Zhejiang. Binary logistic regression was applied to examine the associations between antibiotic misuse behaviours and the social-demographic variables. Analyses were performed with SPSS software (version 20.0).

### Ethical consideration

Written informed consent was obtained from all respondents who participated in the study. It was clearly explained that participation was not compulsory. The study was approved by the Zhejiang University Research Ethics Committee.

## Results

### Socio-demographic characteristics (Table 1)

A total of 2073 university students in Guizhou and 1922 in Zhejiang completed the questionnaires. Students from the two provinces presented very similar social demographics across age, gender, and education level. Medical students accounted for 22.1% (425) of the respondents from Zhejiang University and 14.6% (303) from Guizhou University. Students in Zhejiang were significantly wealthier than those from Guizhou. One-third of students in Zhejiang and two-thirds in Guizhou originated from a rural area. The parents of students in Zhejiang generally had a higher education level, and almost twice as many had a medical background when compared with Guizhou.

**Table 1.** Socio-demographic characteristics of university students in Guizhou and Zhejiang.

	Guizhou (N = 2073)	Zhejiang (N = 1922)	χ^2^/F	*p*
Gender				
Male	1023(49.3%)	1012(52.7%)	4.358	0.04
Female	1050(50.7%)	910(47.3%)		
Age, Mean (SD)	21.3(2.1)	19.7(2.6)	471.1	< 0.01
Education level				
Undergraduate	1696(81.8%)	1561(81.2%)	0.235	0.65
Graduate	377(18.2%)	361(18.8%)		
Major				
Medicine	303(14.6%)	425(22.1%)	37.60	< 0.01
Non-medicine	1770(85.4%)	1497(77.9%)		
Household income per month(RMB)				
< 3000	1099(53.0%)	316(16.4%)	636.3	< 0.01
3000–10,000	794(38.3%)	1092(56.8%)		
> 10,000	180(8.7%)	514(26.7%)		
Hometown				
Rural	1376(66.4%)	702(36.5%)	356.1	< 0.01
Urban	697(33.6%)	1220(63.5%)		
Parents’ highest education level				
Illiteracy/primary school	345(16.6%)	112(5.8%)	464.3	< 0.01
Junior high school	909(43.8%)	459(23.9%)		
Senior high school	464(22.4%)	505(26.3%)		
University and above	355(17.1%)	846(44.0%)		
Parent’s medical background				
Yes	112(5.4%)	176(9.2%)	21.02	< 0.01
No	1961(94.6%)	1746(90.8%)		

### Healthcare-seeking and antibiotic use behaviours (Table 2)

In the past month, roughly 30% of students in both Zhejiang and Guizhou experienced a self-limiting illness. Compared with Zhejiang, students in Guizhou were more likely to see a doctor (35.0% (213) vs. 28.3% (162)) when they were ill, less likely to select primary care health facilities (66.7% (142) vs. 77.1% (125)), and more likely to be prescribed with antibiotics by doctors (79.8% (170) vs. 56.2% (91)). Students in Guizhou were twice more likely to self-treat with antibiotics.

**Table 2.** Antibiotic use of university students in Guizhou and Zhejiang.

Antibiotic use behaviors	Guizhou N (%)	Zhejiang N (%)	χ^2^	*p*
Got illness	609(29.4%)	573(29.8%)	0.091	0.76
Healthcare-seeking behaviours				
Went to see doctors	213(35.0%)	162(28.3%)	12.86	< 0.01
Self-treated	279(45.8%)	254(44.3%)		
Did nothing	117(19.2%)	157(27.4%)		
Prescribed with antibiotics	170(79.8%)	91(56.2%)	24.30	< 0.01
Self-treated with antibiotics	92(33.0%)	41(16.1%)	25.75	< 0.01
Kept antibiotics at dorm/home	1152(55.6%)	1233(64.2%)	30.52	< 0.01
Bought antibiotics without prescriptions	922(73.9%)	578(64.7%)	21.12	< 0.01
Asked for antibiotics	444(21.4%)	300(15.6%)	22.21	< 0.01
Took antibiotics prophylactically	620(29.9%)	302(15.7%)	113.2	< 0.01

In the past year, 55.6% (1152) of students in Guizhou and 64.2% (1233) in Zhejiang kept antibiotics in their dormitory or home. Compared with Zhejiang, Students in Guizhou were more likely to buy antibiotics without prescriptions (73.9% (992) vs. 64.7% (578)) and ask for antibiotics when doctors did not initially prescribe them (21.4% (444) vs. 15.6% (300)), and were almost twice more likely to use antibiotics prophylactically (29.9% (620) vs. 15.7% (302)).

### Determinants of antibiotic misuse (Tables 3 and 4)

After adjusting for all covariates, students in Guizhou were more likely to be prescribed with antibiotics by doctors (OR = 2.95; 95%CI 1.68–5.18; p < 0.001), self-treat with antibiotics (OR = 3.00; 95% CI 1.84–4.90; p < 0.001), buy antibiotics over the counter without prescriptions (OR = 1.71; 95% CI 1.36–2.15; p < 0.001), ask for antibiotics when doctors did not initially prescribe them (OR = 1.48; 95% CI 1.22–1.80; p < 0.001), and take antibiotics prophylactically (OR = 2.28; 95% CI 1.89–2.76; p < 0.001).

**Table 3.** Logistic regression of antibiotic use.

Factors	Went to see a doctor N = 1182 OR (95% CI)	Self-treated with antibiotics N = 533 OR (95% CI)	Prescribed with antibiotics N = 375 OR (95% CI)
Province			
Zhejiang (ref)	1	1	1
Guizhou	1.04(0.77,1.41)	3.00(1.84,4.90)***	2.95(1.68,5.18)***
Age	1.08(1.00,1.17)	1.00(0.87,1.15)	0.91(0.80,1.04)
Gender			
Female (ref)	1	1	1
Male	1.03(0.80,1.32)	1.15(0.77,1.70)	0.80(0.50,1.29)
Education level			
Undergraduate (ref)	1	1	1
Graduate	0.64(0.40,1.01)	0.98(0.49,1.99)	1.79(0.72,4.42)
Major			
Non-medicine (ref)	1	1	1
Medicine	0.78(0.55,1.11)	0.57(0.31,1.02)	0.49(0.26,0.93)*
Education level of parents			
Illiteracy/primary school (ref)	1	1	1
Junior high school	0.74(0.49,1.11)	0.82(0.41,1.66)	1.18(0.55,2.54)
Senior high school	0.82(0.52,1.30)	1.74(0.82,3.73)	1.45(0.62,3.40)
University/above	0.82(0.50,1.37)	1.68(0.73,3.85)	2.57(0.98,6.76)
Parent’s medical background			
No (ref)	1	1	1
Yes	0.65(0.38,1.12)	3.01(1.66,5.47)***	0.86(0.30,2.46)
Household income per month			
< 3000 (ref)	1	1	1
3000 ~ 10,000	0.73(0.53,1.00)	0.65(0.39,1.09)	0.69(0.38,1.26)
> 10,000	0.68(0.44,1.05)	0.66(0.33,1.31)	0.57(0.25,1.30)
Hometown			
Urban area (ref)	1	1	1
Rural area	1.02(0.72,1.44)	0.91(0.53,1.57)	2.01(1.05,3.84)*

*< 0.05; **< 0.01; ***< 0.001

**Table 4.** Logistic regression analyses of antibiotic use.

Factors	Kept antibiotics in dorm/home N = 3963	Bought antibiotics without prescriptions N = 2121	Asked for antibiotics N = 3963	Took antibiotics prophylactically N = 3963
Province				
Zhejiang (ref)	1	1	1	1
Guizhou	1.01(0.86,1.19)	1.71(1.36,2.15)***	1.48(1.22,1.80)***	2.28(1.89,2.76)***
Age	1.00(0.96,1.04)	0.96(0.91,1.02)	1.05(1.00,1.11)*	1.00(0.95,1.05)
Gender				
Female (ref)	1	1	1	1
Male	0.70(0.61,0.80)***	0.96(0.79,1.16)	0.87(0.74,1.02)	0.94(0.81,1.10)
Education level				
Undergraduate (ref)	1	1	1	1
Graduate	0.99(0.78,1.27)	1.94(1.35,2.80)***	1.07(0.81,1.43)	0.83(0.63,1.09)
Major				
Non-medicine (ref)	1	1	1	1
Medicine	1.18(0.98,1.42)	1.15(0.88,1.50)	0.71(0.56,0.90)**	0.69(0.55,0.87)**
Education level of parents				
Illiteracy/primary school (ref)	1	1	1	1
Junior high school	1.33(1.07,1.66)*	0.88(0.63,1.22)	1.10(0.83,1.47)	1.13(0.87,1.46)
Senior high school	1.70(1.32,2.17)***	0.86(0.59,1.24)	1.39(1.01,1.91)*	1.31(0.98,1.74)
University/above	2.03(1.53,2.69)***	0.82(0.55,1.24)	1.40(0.98,2.00)	1.17(0.85,1.63)
Parent’s medical background				
No (ref)	1	1	1	1
Yes	1.68(1.24,2.27)**	0.62(0.43,0.89)*	1.07(0.78,1.48)	1.45(1.08,1.95)*
Household income per month				
< 3000 (ref)	1	1	1	1
3000 ~ 10,000	1.30(1.10,1.53)**	1.14(0.90,1.44)	1.17(0.95,1.43)	0.98(0.81,1.18)
> 10,000	1.14(0.90,1.43)	1.05(0.76,1.46)	1.22(0.92,1.62)	1.03(0.79,1.34)
Hometown				
Urban area (ref)	1	1	1	1
Rural area	0.64(0.54,0.76)***	0.85(0.66,1.09)	1.12(0.90,1.39)	1.10(0.89,1.34)

*< 0.05; **< 0.01; ***< 0.001.

Students with a medical background were significantly associated with better antibiotic use behaviours. They were less likely to be prescribed with antibiotics by doctors (OR = 0.49; 95% CI 0.26–0.93; p < 0.05), ask for antibiotics from doctors (OR = 0.71; 95% CI 0.56–0.90; p < 0.01), and take antibiotics prophylactically (OR = 0.69; 95% CI 0.55–0.87; p < 0.01). However, students whose parents had medical backgrounds demonstrated poorer antibiotic use behaviours. They were more likely to self-treat with antibiotics (OR = 3.01; 95% CI 1.66–5.47; p < 0.001), keep antibiotics in their dorm/home (OR = 1.68; 95% CI 1.24–2.27; p < 0.01), and take antibiotics prophylactically (OR = 1.45; 95% CI 1.08–1.95; p < 0.05), but were less likely to buy antibiotics without prescriptions (OR = 0.62; 95% CI 0.43–0.89; p < 0.05).

Female students, those with higher household incomes, from urban areas, and whose parents had a higher education level or medical background were more likely to keep antibiotics in their dorm/home. Students from rural areas were more likely to be prescribed with antibiotics by doctors (OR = 2.01; 95% CI 1.05–3.84; p < 0.05).

## Discussion

This study investigated the antibiotic misuse behaviours of university students from two provinces that represent a wide diversity of social and economic development in China. We identified that university students in the less developed province were more likely to misuse antibiotics, including asking for antibiotics during a hospital visit, using antibiotics for self-medication, buying antibiotics without prescriptions, and taking antibiotics prophylactically. We also demonstrated significant associations between socio-demographic characteristics and antibiotic misuse behaviours. To our knowledge, this represents the first study investigating the disparity in antibiotic misuse behaviours between well-educated young populations in developed and less developed regions with vastly different levels of social and economic development.

In China, antibiotic misuse has been attributed to factors at both the supply and demand side of the health system. On the supply side, this study showed that the overprescribing of antiobiotics by doctors was more prevalent in the less developed region. Misaligned economic incentive for health providers is the main contributor to the overprescribing of antibiotics in China. Although China has implemented a zero drug profit policy since 2009, which aimed at removing profits on drug sales of health facilities, the connection between drug companies and individual doctors still contributes to this problem [18,19]. Compared with more developed regions, healthcare providers in less developed regions had lower incomes, experienced relaxed regulations, and were more likely to profit from overprescribing antibiotics [7,15,20]. Our findings also indicated that, even when doctors were initially unwilling to prescribe antibiotics, more students pressured doctors to prescribe antibiotics in less developed regions compared to developed regions [16,21].

Pharmacy also represents a supply-side actor in antibiotic overuse. Globally, most antibiotic use occurs outside hospitals, and non-prescription access to antibiotics is common [22,23]. Although the Chinese Food and Drug Administration has had mandatory regulations banning non-prescription purchases of antibiotics for over 10 years, weak enforcement of this policy has enabled easy access to over-the-counter antibiotics in most parts of the country [24]. The higher rate of over-the-counter purchases of antibiotics without prescriptions in Guizhou indicated even poorer supervision and enforcement of regulations in this less developed region.

On the demand side, even while participants in both less and more developed regions are well educated, other socio-demographic characteristics were found to affect antibiotic misuse. Urban students were less likely to be prescribed with antibiotics by doctors than were their rural counterparts. A possible reason for this is that students from rural areas were more easily induced by doctors than were urban students. Previous studies have shown that doctors were much less likely to prescribe antibiotics if patients demonstrated their knowledge of appropriate antibiotic use during the consultation [13,25]. Another finding is that graduate students were more likely to buy over-the-counter antibiotics without a prescription than were undergraduates. The association between self-medication with antibiotics and higher education level has been found by studies in the European and Jordanian contexts [26,27]. Our finding has contributed to the literature that higher education level is a risk factor for antibiotic misuse behaviours. Potential research should be further conducted to examine the effect of education level by exploring the association between education level and demand-side misuse behaviours including self-medication with antibiotics, buying antibiotics over-the-counter without a prescription, and keep antibiotics at home.

In this study, we found that having a medical background represents a protective factor for antibiotic use behaviours. The predominant explanation for this result is that, compared with their non-medical counterparts, medical students generally get more professional knowledge and guidance on appropriate antibiotic use through their (at a minimum) five-year medical curriculum, which usually consists of a series of didactic lectures on antibiotics and opportunities to observe the antibiotic prescribing practices of senior physicians during their internship. A study carried out among university students in northeastern China has supported this finding, and similarities can be drawn from previous studies conducted in Jordan that highlight existing gaps in the knowledge between medical and non-medical students regarding antibiotic use [28,29]. Thus, we recommend that health education programmes aimed at the general public regarding appropriate antibiotic use be implemented, to curb widespread antibiotic misuse behaviours.

Students whose parents had a medical background were associated with higher antibiotic misuse behaviours, including self-treatment with antibiotics, keeping leftover antibiotics in their dorm/home, and taking antibiotics prophylactically. A possible reason for this is that students from a family with a medical background have been self-treated by their parents when they were young, and maintained these habits into adulthood. However, a Korean study found that a higher proportion of physicians and pharmacists believed that antibiotics could treat the paediatric common cold or reduce its complications, when compared with parents, which indicates that healthcare providers may possess higher levels of unfounded beliefs regarding the effectiveness of antibiotics than do laypeople [30].

Keeping antibiotics in the dorm/home has been identified as an important contributor to antibiotic misuse [13]. In our study, students who were female, with a higher household income, who originated from urban areas, and whose parents had higher education levels were more likely to keep antibiotics in their dorm/home. The sources of these antibiotics included non-completed courses of prescribed antibiotics, overprescribing by doctors, and over-the-counter purchase [13]. A former review indicated that over one-third of patients prescribed with antibiotics did not properly comply with their antibiotic therapy, and one-quarter retained leftover antibiotics for future use, which reflects a widespread pattern of poor antibiotic-taking and prescribing behaviours [31]. The problem of leftover antibiotics can possibly be addressed in the following ways: (1) regulate and supervise prescribing practices of doctors so as to restrain overprescribing of antibiotics; (2) educate patients to take antibiotics exactly as prescribed through effective health education programmes; (3) ban access to antibiotics in pharmacies without prescriptions; and (4) encourage patients to discard leftover antibiotics.

Many studies indicated that antimicrobial resistance is increasing in developing countries [23]. As mentioned above, wide variations remain between developed and less developed China, especially in regard to economic development, which determines the general socio-economic status of the local population and exerts significant influence on antibiotic use of the general public. Thus, regional disparities in antibiotic use behaviours represent a valuable area of inquiry. Findings from this study are of considerable concern, especially the indication that antibiotic misuse is more severe in poor areas. Hopefully, results from this study will prompt greater efforts towards public education on appropriate antibiotic use in the less developed region of China.

Another notable finding is that university students lacked adequate knowledge on appropriate antibiotic use, and performed a variety of antibiotic misuse behaviours. Greater attention should be paid to improving the awareness and knowledge of appropriate antibiotic use among this population. University students represent a highly educated group of the population, and their knowledge and behaviour concerning public utilization of antibiotics may significantly influence antibiotic-related issues in China in the future. Furthermore, they represent the next generation of parents in China, and paediatric antibiotic overuse is a pressing concern [24]. Most importantly, public health education on appropriate use of antibiotics and measures to curb overprescribing behaviours by doctors should focus more on less developed regions.

There are several limitations to this study. Firstly, this is a cross-sectional study, and as such cannot establish cause–effect relationships between factors and antibiotic misuse behaviours. Secondly, this study mainly focused on antibiotic misuse behaviours of university students, but did not explore the attitudes or knowledge of students on antibiotic use and social habits of self-medication with traditional herbs in Guizhou and Zhejiang, which turned out to be essential factors in analysing determinants of antibiotic use behaviours. Finally, the survey was conducted in Wen Juan Xing, which makes it difficult to validate the behavioural responses. Future studies using experimental, longitudinal, or case-control designs could help provide evidences for causal relationships. Although the data rely on self-reporting, our survey items were adapted from previously tested surveys. In general, we believe the survey is well representative of the university student population in developed and less developed China.

Obviously, antibiotic misuse for self-limiting illnesses by well-educated young adults was very high in these two sampled regions but most serious in less developed China. A campaign is urgently needed for appropriate prescribing of antibiotics by doctors, enforcing restrictions on selling antibiotics over the counter without prescriptions, and to educate the general public about the management of self-limiting illness.

## Paper context

Antimicrobial resistance is one of the greatest threats to public health in China. The study found antibiotic misuse for self-limiting illnesses by well-educated young adults was very high in the two regions with vast social and economic diversity but most serious in the less developed one. Interventions are urgently needed to promote appropriate antibiotic prescribing by doctors, enforce restrictions on the sale of over-the-counter antibiotics without prescriptions, and to educate the general public about the management of self-limiting illnesses.
